# Prebiotics in food and dietary supplements: a roadmap to EU health claims

**DOI:** 10.1080/19490976.2024.2428848

**Published:** 2024-11-15

**Authors:** Kieran Tuohy, Elaine E. Vaughan, Lucien F. Harthoorn, Ellen E. Blaak, Philip W. J. Burnet, Alessandro Busetti, Anirikh Chakrabarti, Nathalie Delzenne, Paul de Vos, Louise Dye, Damien Guillemet, Lesley A. Houghton, Alwine F. M. Kardinaal, Cath Mersh, Kathy Musa-Veloso, Annegret Nielsen, Justyna Palasinska, Seppo Salminen, Gemma Walton, Naomi Venlet, Charlotte Hubermont, Philip C. Calder

**Affiliations:** a School of Food Science & Nutrition, University of Leeds, Leeds, UK; b Sensus (Royal Cosun), Roosendaal, The Netherlands; c Clasado Ltd, Reading, UK; d Department of Human Biology, NUTRIM, School of Nutrition and Translational Research in Metabolism, Maastricht University Medical Centre+, Maastricht, The Netherlands; e Department of Psychiatry, University of Oxford, Oxford, UK; f Metabolon Inc, Morrisville, NC, USA; g Cargill R&D Centre Europe, Vilvoorde, Belgium; h Woluwe Louvain Drug Research Institute, UCLouvain, Brussels, Belgium; i Pathology and Medical Biology, University Medical Center Groningen, Groningen, The Netherlands; j Institute for Sustainable Food, University of Sheffield, Sheffield, UK; k Nexira, Rouen, France; l Leeds Institute of Medical Research, University of Leeds, Leeds, UK; m NIZO, Ede, The Netherlands; n Cath Mersh Communications, Aarhus, Denmark; o Intertek Health Sciences Inc., Mississauga, ON, Canada; p Analyze & Realize GmbH, Berlin, Germany; q Argenta Barcelona, Barcelona, Spain; r Center for Food and Nutrition Research, Faculty of Medicine, University of Turku, Turku, Finland; s Food and Nutritional Sciences, University of Reading, Reading, UK; t ILSI Europe, Brussels, Belgium; u School of Human Development and Health, Faculty of Medicine, United Kingdom and NIHR Southampton Biomedical, Research Centre, University Hospital NHS Foundation Trust and University of Southampton, Southampton, UK

**Keywords:** Prebiotic, microbiota, biomarker, metabolite, digestive, immune, metabolic, cognitive, health claim, EU, EFSA

## Abstract

Numerous studies have established that prebiotic ingredients in foods and dietary supplements may play a role in supporting human health. Over the three decades that have passed since prebiotics were first defined as a concept, research has revealed a complex universe of prebiotic-induced changes to the human microbiota. There are strong indications of a direct link between these prebiotic-induced changes and specific health benefits. However, at the present time, the EU has not permitted use of the term ‘prebiotic’ in connection with an approved health claim. This paper is the outcome of a workshop organized on the 25^th^ October 2023 by the European branch of the International Life Science Institute (ILSI). It provides an overview of the regulatory requirements for authorized health claims in the EU, key areas of prebiotic research, and findings to date in relation to prebiotics and digestive, immune, metabolic and cognitive health. Research gaps and documentation challenges are then explored and a roadmap proposed for achieving authorization of ‘prebiotic’ in the wording of future EU health claims.

## Introduction

Prebiotics as a concept first gained international attention in 1995, when Gibson and Roberfroid proposed the initial definition.^1^ Since then, the concept has been redefined several times as ongoing research has broadened the understanding of how prebiotics in foods and dietary supplements may benefit health. The consensus definition published by the International Scientific Association for Probiotics and Prebiotics^2^ is the current scientific reference point. ISAPP defines a prebiotic as a substrate that is selectively utilized by host microorganisms conferring a health benefit.

Today, *in vitro* and *in vivo* rodent and human studies have established many possible links between specific prebiotic ingredients and beneficial effects for digestive, immune, metabolic and cognitive health. Yet the European Commission has not approved the specific term ‘prebiotic’ as a health claim. Certain health claims for non-digestible carbohydrates have been authorized, however, some of which include prebiotics as recognized by ISAPP.

This paper provides an overview of the regulatory status of prebiotics in the European Union (EU) and key findings from prebiotic research in relation to health outcomes. Against this background, the purpose is to outline remaining gaps in evidence-based knowledge and propose a roadmap to prebiotic health claims within the EU.

## Methods of this review

This paper is the outcome of a Prebiotic Task Force activity organized by the European branch of the International Life Sciences Institute (ILSI). The topic of ‘Prebiotics and identifying knowledge gaps and a roadmap for building a health claim portfolio’ with a focus on Europe, was developed and debated by all the authors among a larger group of invited scientists at a live workshop on 25^th^ October 2023 in Brussels.

The workshop consisted of presentations given by experts followed by in-depth discussions in a full day programme (https://ilsi.eu/prebiotic-sandpit-programme/). Representatives from academia and industry were invited to contribute.

Briefly it opened with an introduction to prebiotics, microbiota and health, and current regulatory aspects in Europe. Four key health areas were then addressed in separate sessions, namely, (1) digestive, (2) metabolic, (3) immune and (4) cognitive health, followed by a concluding summary session. During the workshop preparation, the organizing committee compiled speaker briefs for the presenters and questions to be addressed. The presenters and chair persons were mainly academics except for some industry representative of regulatory agencies. The topic and questions were discussed by all the attendees of the session. While the invited expert speakers gave overviews on the current state of evidence for these topics, the associated group discussions gave rise to additional knowledge, potentially not addressed during the talks, and to further identify research, technology and regulatory gaps for the specific topics.

This paper comprises summaries of the invited presentations as well as from the session’s discussions which resulted in the final recommendations by the authors.

## What defines a ‘prebiotic’?

**Figure 1.**
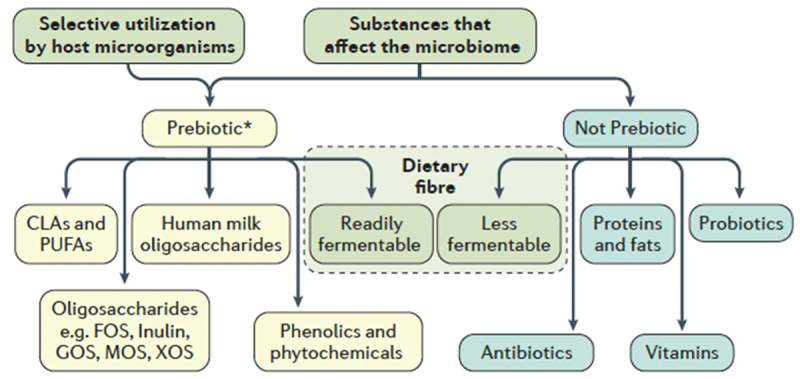
Classification of prebiotics and non-prebiotics. *accepted and candidate prebiotics, as defined by the ISAPP consensus statement, include fructooligosaccharides (FOS), galactooligosaccharides (GOS), conjugated linoleic acids (CLAs), polyunsaturated fatty acids (PUFAs), mannanoligosaccharides (MOS), xylooligosaccharides (XOS). Reproduced from.^2^

The panel that updated the ISAPP consensus definition of prebiotics of 2017 comprised experts in microbiology, nutrition and clinical research. With a view to the latest scientific findings, they agreed the broadest definition of a prebiotic to date: a substrate that is selectively utilized by host microorganisms, conferring a health benefit.^2^ This expanded the concept to include non-carbohydrates, such as polyunsaturated fatty acids and (poly)phenolics (Figure 1), and other target sites beyond the colon. The panel also found that oral administration is not a prerequisite for a prebiotic.

When added to foods and dietary supplements, all prebiotics must be able to resist host enzymatic digestion, ensuring their availability for microbial fermentation by health-promoting microbial groups that often include bifidobacteria and lactobacilli and certain dominant commensal butyrate producers.^2^ The metabolites that result from this fermentation process may be key drivers of potential prebiotic health benefits for the host.^2^ Short-chain fatty acids (SCFAs), for example, are among the metabolites of interest, linked with digestive,^2^ immune,^3^ metabolic^4^ and cognitive^5^ health.

## Current regulatory status in the EU

The EU Nutrition and Health Claims Regulation (Regulation (EC) No 1924/2006) states that any reference to general, nonspecific health benefits of a food or nutrient must be accompanied by an authorized specific health claim (European Parliament 20/12/2006). However, as the regulation does not explicitly define the term ‘prebiotic’ for use as a health claim, EU member states have issued varying advice regarding their tolerance of this and similar terms, such as ‘probiotic’. While this has led to a certain lack of harmonization, most member states agree with the European Commission that use of ‘prebiotic’ in the labeling or advertising of foods and food supplements is an implied health claim and not permitted unless used in conjunction with an authorized health claim.

The European Food Safety Authority (EFSA), which advises the Commission, sets out the requirements that must be satisfied to qualify for an approved health claim. In short, EFSA must issue a favorable opinion based on the answers to the following three questions:^6^

- Has the food/constituent been properly defined and characterised?
- Does the claimed effect have a defined physiological benefit for human health?
- Has a cause-and-effect relationship been established between the consumption of the food/constituent and the claimed effect for the target group under the proposed conditions of use?

EFSA has issued various guidelines for the design, conduct and reporting of studies and clinical trials that provide the necessary documentation for health claim applications.^6^ These guidance documents should be consulted prior to the conduct of any clinical study intended for supporting claims in the EU, as they include important information about the validity of tools used to assess certain outcomes and other requirements such as minimum study length and the appropriate study population.

At present, these guidelines do not propose a roadmap to documenting prebiotic activities. Although prebiotic substances have been shown to support host microorganisms that are both seen as desirable and as producing desirable metabolites, EFSA does not consider this to be sufficient proof of a direct beneficial effect on host health. Prebiotic status depends on sufficient clinical evidence of the selective effect on the microbiota, the microbiota’s role in the proposed health benefit and demonstration of an actual health benefit.

Despite the absence of the term ‘prebiotic’ in the EU register of health claims, a few health claims have been approved for at least one substance widely acknowledged as prebiotic. EFSA has, for example, published an independent favorable opinion on a cause-and-effect relationship between the well-known prebiotic chicory inulin and its contribution to maintaining normal bowel function.^7^

Several other ingredients have EU-approved health claims in relation to lowering blood glucose including non-digestible carbohydrates,^8^ such as fructooligosaccharides (FOS) and galactooligosaccharides (GOS). Non-digestible carbohydrates with an authorized health claim for bowel habit include lactitol^9^ and sugar beet fiber.^10^

The options for making prebiotic health claims appear more straightforward in some other parts of the world – providing clear advantages for food and supplement manufacturers.

This includes Canada, China, Japan and the USA, where the regulatory frameworks have enabled the use of various structure/function claims on health foods and supplements that contain prebiotics. It should be noted, though, that in Japan and the USA some critical voices are questioning the scientific evidence behind some of these claims.^11,12^

Given the lack of harmonized criteria for using ‘prebiotic’ as a term in the EU, individual EU member states have chosen to provide national guidance on the use of ‘prebiotic’ and ‘probiotic’ in the marketing of foods and dietary supplements.^13^ In Italy, for example, the Ministero della Salute tolerates use of ‘prebiotic’ in food and supplement labeling and advertising on condition that the prebiotic is recognized as safe for human consumption within the EU and that there is scientific evidence of efficacy to support the amount added to foods and supplements. It also recognizes an indication of use to ‘promote intestinal flora balance’.^14^ What this trend highlights an overriding issue that has existed ever since the EU Nutrition and Health Claims Regulation was introduced^15^ – the lack of clarity on a research path toward meeting regulatory demands for substantiating prebiotic health claims.^16^

## The state of the art of prebiotic research

ISAPP recently prepared a perspective on the classification of compounds as prebiotics.^17^ Prior to 2017, definitions of the prebiotic concept reflected the shortcomings of early research on the gut microbial ecosystem. This was when references to the selective utilization of prebiotic substances primarily focused on lactobacilli and bifidobacteria. It is now known that the microbiological methods used for this research at that time were not equipped to uncover the full complexity of prebiotic-induced microbial changes.^2^

High-throughput sequencing and molecular analysis technologies have played a key role in identifying additional groups of microorganisms that utilize accepted and candidate prebiotics. Using these tools, scientists have discovered that prebiotic substances may be selectively utilized by one or more microbial groups – conferring a health benefit as a result of that fermentation or metabolic process.^2^ Studies have shown, for example, that oligosaccharides, acacia fiber,^18^ inulin and FOS^19^ stimulate the growth of bifidobacteria in the colon. In addition, it has been found that other candidate prebiotics and prebiotic mixes contribute to an increased abundance of important butyrate producers, including *Faecalibacterium prausnitzii* and *Roseburia* spp. and mucin-degrading, mucosa-fortifying *Akkermansia muciniphila*.^20,21^

Novel technologies are being implemented to demonstrate selective effects, including multiplex community sequencing. This can provide an overview of how a potential prebiotic may affect an entire microbial community and determine which microorganisms are enriched and which may be compromised.^22^

For some indications, a shortage of generally agreed validated biomarkers of intestinal health effects is one of the barriers to a successful health claim application. Today, the emergence of the metabolomic field suggests that more biomarkers could be on the horizon. Metabolomic research has introduced a powerful new toolbox for investigating the metabolic activity that occurs when prebiotics act as substrates for microbial fermentation. Techniques such as ultra-performance liquid chromatography tandem mass spectrometry (UPLC-MS/MS) can provide a detailed, holistic snapshot of microbial responses to nutritional intervention.^23^ Such research opportunities are laying the ground for going beyond mere descriptions of a microbial response to a prebiotic to a portrayal of the functional response and underlying mechanism.^23^ Use of these approaches, such as metabolomics, could support additional data integration (e.g. bridging insights across study cohorts) and interpretation (e.g. understanding microbiota dynamics in response to prebiotic intervention), moving forward and help establish prebiotic properties within the regulatory definitions and health claim acceptance framework by revealing plausible and relevant modes of action to support such a health claim.

Metabolites produced during the microbial fermentation of prebiotic substrates are another major focus of scientific investigation. The choice of such target molecules or biomarkers and when and where to measure them are important considerations. Among them, SCFAs, such as butyrate, propionate and acetate, are of particular interest due to their varied contribution to human health – butyrate and propionate being known for their anti-inflammatory properties and benefits for digestive, immune and metabolic health, while acetate has been linked to energy metabolism and satiety.

Increasing knowledge about the health role of SCFAs could make them a valid biomarker for prebiotic efficacy in the future. At present, quantification and bioavailability evaluation of SCFAs *in vivo* remain a challenge. For example, fecal SCFA concentrations poorly reflect the kinetics of SCFA production, absorption and excretion.^24^ Post-prandial quantification of SCFAs, produced during fermentation of a given prebiotic dose, would also reflect prebiotic fermentation output more clearly than fasting blood samples after chronic ingestion. This is because SCFAs and other nutrient-derived metabolites may be rapidly cleared from the blood. New tools for continuous SCFA measurement before, during and after a meal, therefore, would provide a clearer picture of their effect in individual subjects and enable the design of more robust human studies.

Another area of prebiotic research is investigating the impact of food manufacturing and matrices on prebiotic structure and efficacy. Food manufacturers have the responsibility to ensure that the prebiotic will remain structurally intact and at the appropriate dose for its efficacy following processing into food products or supplements. This will be based upon research often by the food ingredients manufacturer that will advise the final food product manufacturers and perform testing of the prebiotic in the food products as required. Comparisons between studies have often been impeded by the use of different analytical techniques. Therefore, interest was high when the first standardized protocol was used for a study of how food matrices may impact the prebiotic efficiency of inulin-type fructans (ITFs).^25^ While this study largely confirmed previous findings that ITFs are selectively utilized by bifidobacteria regardless of the food matrix, it appeared that prebiotic efficacy was modulated with regard to certain other microbial groups. The use of standardized protocols is of great importance when building documentation for health claim substantiation.

## Prebiotic health benefits – the knowledge so far

Within the scientific arena, prebiotics are recognized as a class of substances that selectively impact the microbial community of a host via their utilization or fermentation. From a health perspective, the consensus panel behind the 2017 ISAPP definition of the prebiotic concept agree on benefits for digestive, immune, metabolic and cognitive health. Bone health in terms of mineral bioavailability is also mentioned.^2^ However, the panel concedes, it remains a challenge to document the relationship between a prebiotic-mediated change in microorganisms (composition or function) and an observed health effect. This is the hurdle that future prebiotic research must overcome.^2^

The next sections provide a status summary of prebiotic research within four health areas: digestive, immune, metabolic and cognitive health.

### Prebiotics and digestive health

EFSA has provided guidance on the scientific requirements for substantiating health claims related to bowel function/gut or digestive health, gastrointestinal (GI) comfort and nutrient digestion/absorption.^26^ Measures of the claimed effect in human studies for gut health and comfort include symptomology assessed using validated global symptom questionnaires, transit time, frequency of bowel movements, stool bulk and stool consistency. Patients with functional constipation or irritable bowel syndrome (IBS) subgroups are considered appropriate study groups for claims on bowel function and GI discomfort.^26^

Several ingredients, prebiotics and candidate prebiotics have attained EU-authorized health claim status within the area of digestive health. These include inulin,^7^ lactitol,^9^ sugar beet fiber^10^ and rye fiber,^27^ which contribute to stool frequency or normal bowel function, and lactulose,^28^ which accelerates intestinal transit. In other words, they are all claims that refer to an improvement in digestive health – as demonstrated in human intervention studies and supported by a plausible mode of action. The absence of an established cause-and-effect relationship is one of the reasons why many digestive health claim applications for candidate prebiotics and other substances have not yet succeeded.

Only in a few cases has EFSA’s scientific opinion made a mechanistic link between the ingredient, the gut microbiota and a digestive benefit. EFSA has found, for example, that the fiber native chicory inulin may exert an effect on stool frequency by stimulating bacterial growth in the gut and, through that, increasing fecal bulk.^7^ Under Article 10.3 of the EC Nutrition and Health Claim regulation, this implies that an ‘inulin prebiotic’ message may be used in conjunction with the authorized bowel habit health claim if all other conditions are met regarding the use of the health claim (European Parliament 20/12/2006). Acceptance of such a message still depends on national authorities. In another scientific opinion, the EFSA panel found that lactulose is broken down by the action of beta-galactosidases from colonic bacteria. This triggers the increase in osmotic pressure and slight acidification of the colonic content that speeds up intestinal transit.^28^

A development of relevance to prebiotic research is that, since 2016, the ROME III definition of Functional GI Disorders (FGIDs) has changed. In light of advances in scientific knowledge of disorders associated with chronic abdominal discomfort and pain and altered bowel function, such as IBS and functional dyspepsia, the Rome Foundation published the ROME IV criteria which replaced FGID with a new term – Disorders of Gut-Brain Interaction (DGBI). ROME IV links DGBI to combinations of five pathophysiological mechanisms: altered gut microbiota, altered central nervous system processing, altered mucosal and immune function, visceral hypersensitivity and motility disturbance.^29^ These and future updates to the criteria should be considered when enrolling patients for clinical trials.

Among IBS patients and others with digestive health conditions, the limited efficacy of drug treatments has inspired a growing interest in dietary management of the symptoms. As the gut microbiota may be implicated in DGBI, potential exists to correct such imbalances and improve host health through prebiotic intervention. Various studies have, for example, evaluated the impact of dietary supplementation with GOS on the gut microbiota.^22^ One finding is that GOS consumption specifically increases the abundance of bifidobacteria in healthy adults, although with considerable variation among individuals.^22^ *In vitro* studies have also found that FOS and acacia-derived arabinogalactan have a positive impact on specific species such as *Faecalibacterium prausnitzii*, which may benefit the gut barrier and inflammation.^30^ A clinical study of acacia fiber recently confirmed this potential, finding that supplementation significantly improved stool frequency in patients with IBS.^31^ Nevertheless, randomized controlled trials (RCTs) have yet to produce sufficient convincing evidence that prebiotics are a beneficial nutritional strategy for relieving IBS symptoms.^32^

Another focus of digestive health studies are the SCFAs and other metabolites generated during the microbial fermentation of prebiotic substrates. Butyrate, for instance, is a particularly important fuel for the colonocytes and, if in short supply, may contribute to an impaired GI function. Other metabolites may play a role in peristalsis, affecting colonic motility.^33^ In some individuals, it is possible elements of the gut microbiota that affect cognitive health may also relieve IBS symptoms. These are all aspects that deserve further research attention to understand how prebiotics can improve digestive health.

### Prebiotics and immune health

Immune health claims recognized by EFSA fall into two categories: defense against pathogens and a beneficial change in response to allergens. In their scientific guidance, EFSA states a requirement for well-controlled human intervention studies that show a relevant clinical effect.^26^ In the case of claims related to defense against pathogens, such effects include a reduction in the incidence, duration or severity of symptoms at a specified site of infection, for example the GI tract, respiratory tract, lower urinary tract or the vagina. Importantly, if there is sufficient scientific evidence that a clinical infection is imminent due to the presence of a particular microorganism and/or its toxin at a particular site of the body, then microbiological data can be used in place of clinical outcomes related to infection. For claims related to beneficial changes in response to allergens, studies must show a reduction in the incidence, duration and/or severity of allergic manifestations in individuals who are at risk but free of symptoms at baseline.

Prebiotics are of interest from an immune perspective because they exert their actions in the gut, where between 70% and 80% of human immune cells reside in the GI tract wall.^34^ As the largest immunological organ, the gut is a central site of immune interactions and immune training in respect of tolerance and defense. It is, therefore, a logical assumption that nutrition and the gut microbiota have an influence on immune health.

Receptors enable the sensing capability of the immune system. The best studied are the toll-like receptors (TLRs), which play a role in mediating immune/inflammatory pathways in the gut. Several studies have found that candidate prebiotics such as pectin, a soluble fiber, may modulate the immune system directly by binding to TLRs^35^ – a non-prebiotic effect as it is not driven by changes to the microbiota. *In vitro* and animal studies indicate, for example, that pectin’s inhibition of TLR-2 could prevent chemotherapy-induced intestinal inflammation.^36^

Observations of the influence of certain dietary fibers and prebiotics on immunity note an enhanced production of SCFAs and other metabolites during microbial fermentation.^35^ *In vivo* research with inulin-type fructans has further demonstrated the possibility of an immune modulating effect both with and without microbiota involvement, depending on fructan chain length.^37^

Important issues must be resolved to document prebiotic benefits for human immune health and, on that basis, build a substantiated case for a health claim. A particular complicating factor is that immune functions vary between individuals and are age and sex-dependent. As yet, EFSA has accepted no validated biomarkers of the immune effect of dietary intervention.^38^ The sole exception is vaccination trials that show an increased antibody titer in excess of a pre-established threshold value known to confer protection against the infection – in other words, an increase in vaccination responders.

An expert panel convened by ILSI Europe has attempted to overcome the biomarker hurdle by developing a stepwise approach to selecting markers for trials and interpreting outcomes.^38^ Interestingly, other regulatory bodies, including Canada’s Natural and Non-prescription Health Products Directorate, reference this stepwise approach when determining the sufficiency of immune claim evidence.

Study populations for clinical trials are typically healthy individuals or individuals at risk of immunosuppression, such as people who are elderly, stressed or engaged in heavy physical exercise. Immune responses may be measured following controlled exposure to a microbe, vaccination against disease or a natural infection, for example during the cold and flu season.

Such clinical studies have produced a series of interesting outcomes. In one challenge trial, carrot-derived rhamnogalacturonan-I was shown to have a protective effect against common cold symptoms. The dual mechanism proposed includes direct interaction with TLRs and microbial fermentation.^39^ In healthy adults, dietary supplementation with inulin-type fructans has also been seen to have a modest influence on the antibody response to a seasonal influenza vaccination^40^ and hepatitis B vaccination.^41^

Changes in the gut microbiota are known to accompany the aging process, including a decline in bifidobacteria. This has focused attention on the ability of prebiotics to reverse such a decline and, through that, improve the immune response in elderly individuals. In this regard, GOS has shown promise as a prebiotic supplement for enhancing the microbial and immune systems.^42^

Despite the published studies that indicate the benefits of (candidate) prebiotics for the immune system, this evidence is still insufficient to meet scientific substantiation requirements for a health claim in the EU. To facilitate progress, an ILSI Europe expert group is currently evaluating the documented effects of prebiotics on immunity, inflammation and infection, obtained from RCTs in humans.

### Prebiotics and metabolic health

Global obesity has tripled since 1975,^43^ creating the need to define more strategies for improving metabolic health and reducing obesity-associated comorbidities. Today, the link between obesity, insulin resistance, cardiometabolic risk factors and an altered gut microbiota is widely recognized,^44^ with diet a core element. This has provided the rationale for studying how dietary fibers may contribute to metabolic health through the action of the gut microbiota modulation.^44^ At present, a clear cause-and-effect relationship has not been established between a prebiotic-driven change in the microbiota and improved insulin sensitivity, blood pressure and other metabolic health indicators.

ITFs have been the focus of numerous, primarily animal, studies, which have found a modulating effect on obesity and metabolic disorders.^45^ It has been suggested that metabolites generated during the fermentation of ITFs, including the SCFAs acetate, propionate and butyrate, may contribute to appetite regulation, insulin secretion and intestinal transit.^46^

The FOOD4GUT project in Belgium has investigated the effect of ITFs in clinical trials with healthy individuals and individuals living with obesity. Healthy adults who consumed a diet high in ITF-rich vegetables experienced increased satiety and a reduced desire to eat sweet and salty food.^47^ This coincided with a 3.8-fold increase in the *Bifidobacterium* genus and a reduction in unclassified Clostridiales – an increased level of Clostridiales having previously been connected with a high-fat diet in rats.^47^

In a trial in people living with obesity, the combination of ITF-rich vegetables and an inulin supplement led to reduced nutrient intake, weight loss and specific modifications of the gut microbiota. The microbiota modulation was, however, considerably less pronounced in those subjects who received metformin as a diabetes treatment.^44^ Because metformin has a known impact on the gut microbiota, these findings demonstrate how the microbiota’s baseline composition can impact prebiotic efficacy.^44^ Further research has shown that the efficacy of an inulin-enriched diet may be improved when combined with physical exercise.^48^

One important question concerns the site of the prebiotic effect in the GI tract. Do SCFAs have a greater impact on metabolic health if they are made available in the proximal colon or the distal colon^4^? In one trial that investigated the effects of SCFA acetate infusions in men living with overweight or obesity, distal colonic infusions gave the most significant improvement in metabolic markers.^4^ A follow-up trial showed a series of metabolic effects following rectal administration of SCFA mixtures, suggesting that potential exists for improving body weight control and insulin sensitivity.^49^ The SCFA mixtures used were in concentrations equivalent to those that could be realistically obtained following fiber consumption. A complex fiber structure, comprising chicory root inulin with resistant potato starch, in an acute trial enabled SCFA delivery to the distal colon for fermentation and has shown marked effects on human metabolism and metabolic markers.^50^ Notably, these effects were only observed in lean individuals and not individuals with overweight and prediabetes – it is possible that longer consumption of the SCFA mixtures may be required to modify the gut microbiota of overweight persons in order to observe similar metabolic effects.

Findings that link dietary fibers and certain prebiotics to metabolic health are in harmony with the EFSA recommendation for a high-fiber diet. However, more knowledge of the gut microbiota and the modulating effect of SCFAs is necessary to define specific prebiotic benefits for metabolic health in different metabolic subgroups.

EFSA has outlined requirements for studies that could lead to a metabolic health claim in several guidance documents.^51,52^ Acceptable outcomes in the area of body weight/composition include weight loss, body fat loss, increased/maintenance of lean body mass, body weight maintenance after weight loss, and improved glycemic and insulinaemic responses.^51^ In relation to cardiac function, examples of acceptable outcomes are beneficial changes in blood lipid levels in the long-term or post-prandial reductions in triglyceride levels; improvements in arterial blood pressure and the elastic properties of the arteries, endothelial function or venous blood flow; and reductions in platelet aggregation or homocysteine levels.^52^ Of note, EFSA recognizes LDL cholesterol as a risk factor in the development of coronary heart disease, and systolic blood pressure as a risk factor in the development of coronary heart disease and stroke.

### Prebiotics and cognitive health

Over the past decade, studies of the gut-brain axis have produced increasing evidence that the gut microbiome is associated with psychiatric and cognitive dysfunction.^5^ This has inspired growing interest in probiotics as a means to reducing cognitive deficits and enhancing cognitive function in general. While research findings have documented probiotic effects on cognition, the mechanisms behind these effects remain poorly understood^5^ as many of these studies have been conducted in animal models or in vitro fermentation models, or they employed cognitive tests that lack sensitivity to nutritional manipulation.^53^

One proposed mechanism is that probiotic effects occur through alterations in microbial metabolites, including SCFAs.^53^ This proposal could implicate prebiotic substrates that support microbial fermentation, but, at present, there is little evidence of which type or dose of prebiotic delivers the most efficacious cognitive effect.

Animal trials with prebiotics have explored various aspects of cognitive function. A 10-week prebiotic intervention with topinambur powder and chicory root inulin was found to mitigate the negative effects of mild, unpredictable stress on cognition and intestinal dysbiosis.^54^ Post-natal intake of GOS has been seen to reduce anxious behavior in rats, possibly through the reduction of stress-related gut bacteria.^55^ In a mouse model of Alzheimer’s disease, mannanoligosaccharides reduced cognitive and behavioral deficits, an effect partly attributed to a remodified microbiome and enhanced SCFA formation in the gut.^56^

Tests of cognitive flexibility in animals map similar processes in the developed human brain. Building on findings of improved cognitive flexibility in rats following GOS intake,^57^ a human clinical study showed that GOS consumption produced similar cognitive benefits in medicated psychosis patients.^58^ These results suggest that findings from animal prebiotic trials may be translated to humans; however this would have to be shown in RCTs.

Among the comparatively few clinical studies of prebiotics and cognitive function, other studies also stand out. A diet rich in prebiotic and fermented foods has been seen to reduce perceived stress in healthy adults, although with only subtle changes in microbial composition and function.^59^ One of the conclusions from this particular study was that habitual diet may have a bigger impact on the gut microbiota than a short-term intervention. A more noticeable impact on microbial composition was noted in another study of healthy, working adults. Here, consumption of oligofructose and 2’fucosyllactose produced a substantial improvement in mood along with a simultaneous increase in *Bifidobacterium*, *Roseburia*, *Faecalibacterium* and *Prevotella*.^20^

Surprisingly few studies have examined the potential of prebiotics to improve cognitive function in older adults, a population with known vulnerability to cognitive decline and a greater variability in gut microbiota than younger adults. As such, older adults are a promising target for future studies of how nutritional interventions may benefit cognition. A recent study demonstrated that the 12-week intake of prebiotic ITFs in healthy twins aged 60 years or above resulted in improved cognitive function, particularly in relation to associative learning and memory.^60^

A prerequisite of cognitive function research is that scientifically validated tests are used as markers of specific cognitive outcomes. These markers should be aligned and standardized to enable reliable comparisons of intervention studies. To support this, an ILSI Europe expert panel has set out a series of criteria for validating and selecting appropriate tests of cognitive function.^61^

An ILSI Europe expert group on prebiotics and cognition has written a perspective paper that makes recommendations for future research. They suggest targeting suboptimal cognitive function in healthy individuals, caused by stress, poor sleep, sedentary behavior characterized by little physical exercise, and unhealthy dietary patterns, to define windows of opportunity over a lifetime. Furthermore, they highlight the importance of assessing relevant biomarkers and potential mechanisms of action to identify successful prebiotic interventions in terms of type, dose, timing and duration.^62^

EFSA also provides guidance on the scientific substantiation of claims related to cognitive function, for example, alertness, attention and memory.^63^ This states that well-controlled clinical studies must use valid psychometric tests and that the study group must be generalizable to the target group for whom the claim is intended. In general, studies in subjects with mild cognitive decline but free of dementia or other neurological diseases at baseline are appropriate for extrapolation. Studies in subjects with neurological diseases are considered by EFSA on a case-by-case basis, depending on whether the mechanism is likely to be similar in subjects with and without the disease.

## Research gaps and documentation challenges

Research has documented the potential of prebiotics to enhance animal and human health. However, many questions remain concerning the modulating effect on the gut microbiota and microbial functionality. A continued effort is required to understand mechanisms of action, the relationship between prebiotic structure and function, and how that function results in a health benefit for the host.

Gut microbiota composition and functionality are often measured in feces as a proxy, because specific sites in the GI tract are difficult to access. A lack of noninvasive sampling tools hampers the understanding of spatial and temporal changes induced by prebiotics along the GI tract. Nevertheless, fecal samples are still valid for analysis as long as sample collection and processing follow high standards of rapid processing and appropriate storage conditions prior to further analysis).

Variations in gut microbiota composition and functionality between individuals may be associated with differing responses to (dietary) interventions, as reviewed in.^64^ The initial microbial profile has been found to predict outcomes following dietary fiber interventions,^65^ fecal transplantation^66^ or bariatric surgery.^67^ Additionally, microbial responses to fiber-specific interventions have identified both responder and non-responder phenotypes, which are linked to the levels of SCFAs produced from fiber.^68^ An effect may also be specific to a particular prebiotic or prebiotic dose. In the existing literature, limited attention has been paid to confounding factors known to influence the microbiota, such as diet, body weight, age, host genetics, metabolic phenotype, medicine use and geographical location. Improved knowledge of these aspects would both support the documentation of prebiotic mechanisms and, in the long-term, contribute to building the capability to predict intervention outcomes.

Due to a shortage of standardized tools, clinical studies employ a range of methodologies that often make their findings difficult to compare. While certain prebiotics have been studied more than others, no consensus exists regarding the appropriate amount of a specific prebiotic, the duration and timing of a prebiotic intervention or intervention conditions, except where there is an authorized health claim.

There is also a pressing need for more validated biomarkers of beneficial health outcomes linked to prebiotics, such as immunological changes, inflammatory mediators, serum lipid levels and measures of cognitive function.^2^ Advanced analytical methods are another necessity to extract information from the millions of data points that make up the gut microbiota. Multi-omic technologies provide some opportunities for assessing and quantifying microbial changes,^2^ but there is still a need for *in vivo* sampling tools for various GI locations.

Above all, progress toward establishing direct links between a prebiotic and host health depends on investments in more clinical studies with a robust design. These studies should include cause-and-effect aspects to link changes in the gut microbiota or their metabolites with a physiological function. Although studies have made plausible observations with respect to some prebiotics, such as the effect of dietary fiber on regular bowel movement, EFSA has frequently criticized the limited availability of clinical evidence concerning the mode of action of less well-researched ingredients. Both a clearer interpretation of existing evidence and more *in vitro* and *in vivo* studies are required to address this, including RCTs that focus on the target population which may be healthy study populations and/or subjects with an increased risk of disease.

## A roadmap to EU health claims

EFSA has made it clear that documentation of a prebiotic-driven change in the microbiota must provide direct evidence of a physiological benefit that can be measured in vivo in humans. The high bar is currently a major barrier to integrating the term ‘prebiotic’ in a specific health claim on a food or dietary supplement, though this applies to all health claims.

Due to the many variables that influence the outcome of prebiotic intake, there could be more to gain from highlighting prebiotic activity as an additional benefit of a specific health effect. This could be achieved with reference to Article 10.3 of the EU Nutrition and Health Claim Regulation, when selected members of the microbiota are involved in the health effect and the substance complies with the ISAPP definition of a prebiotic. In the case of a well-recognized prebiotic like inulin, for instance, the EFSA opinion on stool frequency^7^ suggests that it may be possible to incorporate a prebiotic message in claim wording using Article 10.3 (European Parliament 20/12/2006).

Consistent health outcomes from multiple clinical studies may create an opening for consideration.

Based on EFSA’s published guidelines^7^ and the current status of prebiotic research, a roadmap may be proposed toward future authorization of ‘prebiotic’ in the wording of EU health claims.

1. CharacterisationPrebiotic substances should be well defined chemically and their selective effect on the microbiota characterized in detail under realistic *in vitro* and *in vivo* conditions using state- of-the-art methods.
2. Demonstration
  Selective modulation of the microbiota should be associated with a demonstrable physiological benefit and linked mechanistically to that benefit.
3. Documentation
  Multiple clinical studies should document the cause-and-effect relationship between the selective prebiotic effect on the microbiota and the physiological benefit in the target population.

For a well-substantiated health claim application, at least two studies are required to investigate the conditions of use, such as the dose required in a food product or supplement to obtain the claimed effect. The documentation should both show that the prebiotic is bioavailable at the site of microbial fermentation and provide evidence of a plausible mode of action. Standardised protocols, validated biomarkers and advanced data integration and analysis tools are in urgent need to support robust study designs for this purpose.

**Figure 2.**
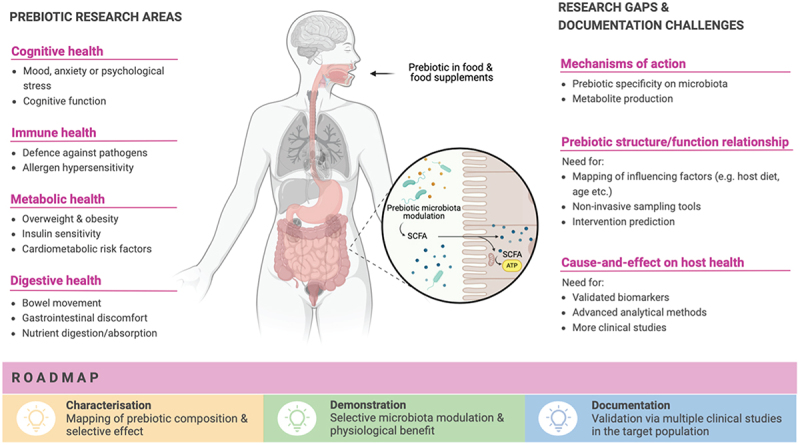
Prebiotic implication in health benefits and roadmap to a related health claim in the EU.

The ambition is to build a health claim dossier that uses the term ‘prebiotic’ in association with a health benefit in Europe (Figure 2). Although the current literature is already extensive, there are still many challenges to overcome to provide appropriately substantiated evidence of prebiotic mechanisms. The need to single out and document specific health benefits through high-quality, comparable trials is indisputable.
